# Fungicidal PMMA-Undecylenic Acid Composites

**DOI:** 10.3390/ijms19010184

**Published:** 2018-01-08

**Authors:** Milica Petrović, Debora Bonvin, Heinrich Hofmann, Marijana Mionić Ebersold

**Affiliations:** 1 Powder Technology Laboratory, Institute of Materials, Ecole Polytechnique Fédérale de Lausanne, 1015 Lausanne, Switzerland; petrovicmilica21@gmail.com (M.P.); debora.bonvin@gmail.com (D.B.); heinrich.hofmann@epfl.ch (H.H.); 2 Faculty of Medicine, University of Nis, 18006 Niš, Serbia

**Keywords:** undecylenic acid, PMMA, antifungal, fungicidal, anti-*Candida*, *Candida albicans*

## Abstract

Undecylenic acid (UA), known as antifungal agent, still cannot be used to efficiently modify commercial dental materials in such a way that this affects *Candida*. Actually, issues with *Candida* infections and fungal resistance compromise the use of Poly(methyl-methacrylate) (PMMA) as dental material. The challenge remains to turn PMMA into an antifugal material, which can ideally affect both sessile (attached) and planktonic (free-floating) *Candida* cells. We aimed to tackle this challenge by designing PMMA-UA composites with different UA concentrations (3–12%). We studied their physico-chemical properties, the antifungal effect on *Candida* and the cytotoxicity toward human cells. We found that UA changes the PMMA surface into a more hydrophilic one. Mainly, as-preparation composites with ≥6% UA reduced sessile *Candida* for >90%. After six days, the composites were still efficiently reducing the sessile *Candida* cells (for ~70% for composites with ≥6% UA). Similar results were recorded for planktonic *Candida*. Moreover, the inhibition zone increased along with the UA concentration. The antifungal effect of UA was also examined at the surface of an UA-loaded agar and the minimal inhibitory concentration (MIC90) was below the lowest-studied 0.0125% UA. Furthermore, the embedded filamentation test after 24 h and 48 h showed complete inhibition of the *Candida* growth at 0.4% UA.

## 1. Introduction

Fungal infections are recognized as one of the major health issues with about 1.2 billion people suffering from it (as reported in 2012) [1,2] and about two million people dying of it every year [3]. *Candida* is among the four fungal species that are mostly causing these mortalities [1]. Among numerous *Candida* species, one of the most found in clinical cases is *Candida albicans* (*C.a.*) [4] and thus, a lot of effort has been devoted to combat *C.a.* [4,5,6,7,8,9,10,11,12,13,14,15].

Although issues with the *C.a.* pathogenicity have been known for a long time, the current state-of-the-art clinical treatments, i.e., mainly the treatment with echinocandins and fluconazole, still cannot combat candidiasis (for instance, the mortality from systemic candidiasis still remains ~50%) [1]. This issue especially affects dentistry where more than 70% of patients suffer from denture stomatitis typically associated with *C.a.* [16]. With that respect, the biggest issue is the formation of *C.a.* biofilms composed of multiple cell types (i.e., round yeast cells, oval pseudohyphal cells, and elongated, cylindrical hyphal cells) enclosed in an extracellular matrix [6]. In fact, among the numerous factors which contribute to the pathogenicity of *C.a.*, the mostly studied ones, which are also recognised as the most critical ones, are the following two: (i) the adherence of yeast-form cells to a surface and (ii) the ability to switch between yeast and hyphal growth forms (dimorphism) [10,17]. Therefore, there is an urgent need for the development of materials that would ideally simultaneously affect both attached to the surface (sessile) and free-floating (planktonic) *C.a.* cells, and inhibit both the adherence of the yeast-form cells to a surface and the yeast-to-hyphae transition [6,10,13,17,18].

One of the fungicidal substances known for decades for its potential to combat fungus is undecylenic acid (UA) [19]. The medical use of this monounsaturated fatty acid, which is naturally occurring even in the human body, is often impaired by its oily nature, thus by its limited solubility and the issues with irritation, taste and odor [20]. Although the antifungal effect of UA has been recognized for a long time, the mechanisms of action of UA on *C.a.* were not known until one recent detailed study in that direction, which found that UA reduces the hypha-to-yeast ratio (even at 1 millimolar), causes the deformation of the surface of *C.a.* cells (at one millimolar) and inhibits numerous other factors, such as hyphal formation, adhesion, mitochondrial activity, cell proliferation, transcriptional regulation of the cell membrane virulent factors, and biofilm formation [21]. Knowing this exceptional potential of UA against *C.a.*, it is of interest to use UA as a filler in polymers, which are often used in implants for medical applications, especially in dentistry. In that respect, the main challenge is to achieve that UA, which is incorporated into such a polymer, gets released in the amount sufficient to show an ultimately simultaneous anti-*Candida* effect on both sessile and planktonic cells.

In the present study, we developed composite materials by incorporating UA into a commercial cold-polymerized acrylic resin Poly(methyl-methacrylate) (PMMA) widely used in biomedical applications, especially in dentistry [22]. The properties of the obtained PMMA-UA composites with UA concentrations ranging from 3 to 12% (*w*/*w*) were studied and compared to the PMMA only (sample 0% UA). To study the influence of UA on the physico-chemical properties of the composites’ surface, the surface of the composites was characterized by Fourier transform infrared spectroscopy (FTIR), by measuring the water contact angle and by high resolution scanning electron microscope (HR SEM). In order to evaluate the potential use of PMMA-UA composites as a fungistatic and fungicidal materials, we examined the morphology of sessile *C.a.* cells on the composites’ surfaces by HR SEM and measured at two timepoints (after the composites’ preparation, T_0_, and six days after their preparation, T_6_): the inhibition zone, the percentage of metabolically active both sessile and planktonic *C.a.* cells, and their toxicity towards human cells. Moreover, we investigated the effect of UA on the morphology and growth of sessile *C.a.* cells in embedded conditions, as well as after the incubation of *C.a.* (at two different concentrations) with agar loaded with different concentrations of UA to find the minimal inhibitory concentration (MIC).

## 2. Results and Discussion

### 2.1. Physico-Chemical Properties of PMMA-UA Composites

**Figure 1:**
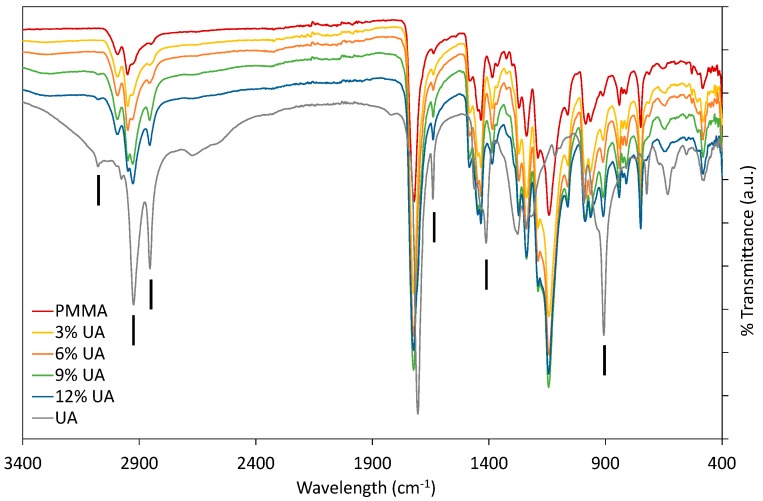
Fourier-transform infrared (FTIR) spectra of composites with 3%, 6%, 9% and 12% (*w*/*w*) of undecylenic acid (UA) compared to native components, Poly(methyl methacrylate) (PMMA) and UA. Black vertical lines indicate peaks characteristic for UA, which were not characteristic of PMMA.

Firstly, PMMA-UA composites were studied by FTIR by comparing the spectra of native components (UA and PMMA) with composites containing different UA concentrations (Figure 1). As expected, composites had all the characteristic peaks of PMMA. Typically the characteristic bands of the carboxylic group are in the 1570–1610 cm^−1^ region for its antisymmetric vibrations, and in the 1350–1450 cm^−1^ region for its symmetric vibrations [23]. However, some peaks of PMMA and UA are similar, because of the similarity of some bonds, as for instance C=O and C–O. Therefore, the presence of UA in the PMMA-UA composites was indicated by the characteristic peaks of UA, which were not present in PMMA, such as: C=C (at 1642 cm^−1^ and 908 cm^−1^), =C–H (at 3078 cm^−1^) and O–H (at 1411 cm^−1^). Both characteristic C=C peaks of UA were evident in PMMA-UA composites, confirming the presence of UA in the composites. Nevertheless, the =C–H bend of UA was visible in the composites with 9% and 12% of UA only as a week peak shifted towards lower wavelengths (3074 cm^−1^), while it was not seen in composites with 3% and 6% of UA. This indicates the interaction of the unsaturated UA tail with PMMA, probably via hydrogen bonding, because C=C peaks were clearly visible. However, the O–H peak of UA at 1411 cm^−1^ was not observed in the composites, suggesting the interaction of this part of the carboxylic group with PMMA. Moreover, a broad peak with the appearance of multiple peaks in the 3300–2500 cm^−1^ region (which corresponds to both O–H and various C–H vibrations) was observed in both PMMA and UA. However, the CH_3_ peak is more characteristic of PMMA (see peaks at 2985 cm^−1^ and 2964 cm^−1^), while CH_2_ is more characteristic of UA, due to a long C_11_ chain (see peaks at 2927 cm^−1^ and 2854 cm^−1^) [24]. All four peaks were observed in PMMA-UA composites, and as expected UA-related peaks were more pronounced in the composites with higher UA concentrations (9% and 12%).

**Figure 2:**
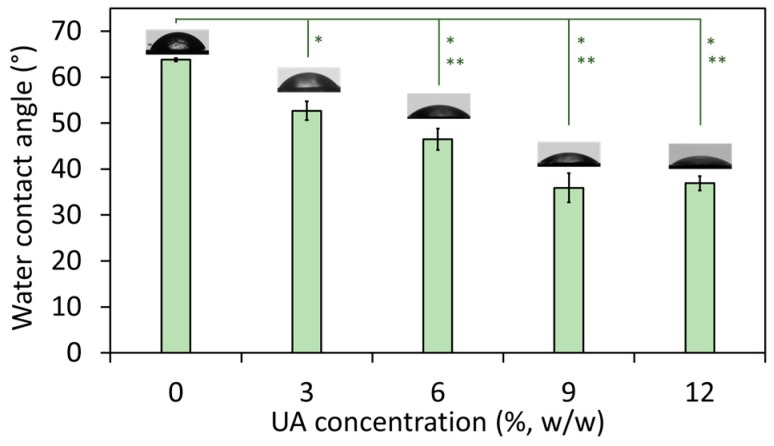
Water contact angle on the surface of composites with the indicated percentage of undecylenic acid (UA) from 0% to 12% (all values are given as mean ± standard deviation). The difference between composites with 3%, 6%, 9% and 12% of UA as compared to Poly(methyl methacrylate) (PMMA) (0% UA) was significant at *p* = 0.05 (*) and *p* = 0.01 (**).

The wetting properties of the composites were studied by measuring their water contact angle. It has to be noted that all studies were done on the native (non-polished) composites’ surface and hence, there are no parallel lines and cracks characteristic for polished PMMA (therefore, polished PMMA has water contact angle ~90° [25]). The results (Figure 2) showed that with an increase of the UA concentration, the water contact angle decreased from 63.8° (for composite with 0% UA) to ~36° (for composites with 9% and 12% of UA). This confirms a change in the surface nature of the studied materials, which become more hydrophilic with an increase of the UA concentration in the composites. The result for PMMA is in agreement with previously reported values (such as ~68° [26]), which has a more hydrophobic nature due to the presence of polar side-chain groups [26]. However, in PMMA-UA composites, we expected that carboxylic groups of UA molecules would be oriented towards the side-chain groups of PMMA, while the nonpolar flexible part of the UA tail would be oriented outwards the PMMA-chain and thus outwards from the surface of the composite. In this way, an increase of the UA concentration would cause an increase of the number of nonpolar UA tails at the composites’ surface. Consequently, the top most layer of the surface of the PMMA-UA composites would appear as more hydrophilic as compared to the PMMA surface. This effect should be more pronounced with an increase of UA concentration. Such a behavior is indeed observed for composites with UA concentrations from 0% to 9%. However, the water contact angle in-between composites with 9% and 12% of UA did not change. This suggests that the presence of UA on the surface of composites was not the only reason for the observed increased hydrophilicity.

**Figure 3:**
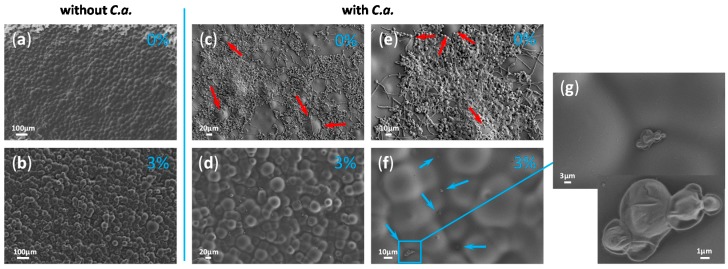
Micrographs obtained by high resolution scanning electron microscope (HR SEM) of the surface of Poly(methyl methacrylate), PMMA, (**a**,**c**,**e**) and composite with three percent of undecylenic acid (UA) (**b**,**d**,**f**,**g**) both without incubation with *Candida albicans* (*C.a.*) (**a**,**b**) and with *C.a.* incubation (**c**–**g**). HR SEM micrograph in (**g**) is a magnified region from (**f**) indicated by the blue rectangle. Red arrows in (**c**,**e**) point towards protrusions on the surface not occupied by *C.a.* cells, while blue arrows in (**f**) point to valleys on the surface in which rare *C.a.* cells could be observed.

**Figure 4:**
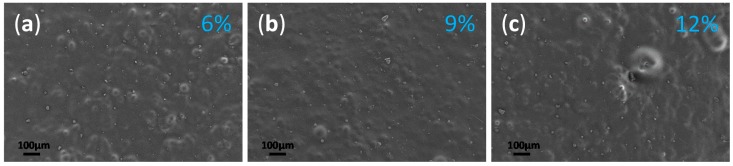
Micrographs obtained by high resolution scanning electron microscope (HR SEM) of the surface of the composite with: (**a**) 6%, (**b**) 9% and (**c**) 12% of undecylenic acid (UA) after their incubation with *Candida albicans* (*C.a.*) cells.

In fact, besides the chemical groups at the materials’ surface, other factors, such as the surface topology, influence the hydrophilic/hydrophobic appearance of that surface. For instance, the surface with convex structures (ideally periodic) would appear as hydrophobic (the phenomenon called lotus effect), while the smooth non-structured surface behaves as hydrophilic. Therefore, we examined the surface of the studied composites by HR SEM. In fact, it has been often reported that a polished PMMA surface is either rough or contains voids due to the lines and cracks from the polishing process [25,27,28]. On the contrary, non-polished PMMA does not have lines and cracks and indeed, HR SEM micrographs of the native non-polished PMMA surface showed a smooth but not-ideally-flat surface (Figure 3a). More precisely, the PMMA surface has a wavy topology and this is an additional reason for the more hydrophobic nature of the PMMA’s surface besides the polar side-chain groups. The surface of the composites with three percent of UA was also non-flat, but more irregular in the surface profile than PMMA (Figure 3b). However, composites with 6%, 9% and 12% of UA had similar surfaces (see for instance Figure 4), which were flatter than those of PMMA and the composite with 3% UA. Only a small difference could be observed in-between the composites with 9% and 12% of UA. Namely, the composite’s surface with 12% UA appeared with a slightly irregular topography and surface protrusions. Since this would increase the hydrophobicity, while an increase of the UA concentration would increase the surface hydrophilicity, it is possible that the compensation of these two opposing effects accounts for the same water wetting angle in-between composites with 9% and 12% of UA (see Figure 2).

### 2.2. Anti-Candida Properties of PMMA-UA Composites

The surface of all composites was also studied by HR SEM after 24 h incubation of the composites with *C.a.* Particular attention was given to the composites with zero percent and three percent of UA, because of the above observed relatively small differences in both the water contact angle (Figure 2) and the surface topography (Figure 3a,b). Therefore, these two composites were examined after their incubation with *C.a.* A large difference could be observed between the PMMA surface almost fully covered by *C.a.* (Figure 3c) and the surface of the three percent UA composite almost without *C.a.* cells (Figure 3d). Interestingly, HR SEM micrographs with a higher magnification of the *C.a.* biofilm on the native PMMA surface (Figure 3e) show that *C.a.* cells (typically as true hyphae) firstly grow in the “valley” by leaving “protrusions” unoccupied (see red arrows in Figure 3c,e). This was also confirmed by the HR SEM micrographs with a higher magnification of the *C.a.* cells on the surface of the composite with three percent UA (Figure 3f), which shows that rare *C.a.* cells (typically as yeast) were firstly found in the “valley” (see blue arrows in Figure 3f and a micrograph of a higher magnification in Figure 3g of the region marked in blue in Figure 3f). In other words, it seems that *C.a.* cells prefer to attach to all kinds of concave structures, such as holes and valleys. This is in agreement with previous reports of the tendency of *C.a.* to attach to holes [28]. These observations suggest that the hydrophobic nature of some materials attracts *C.a.* cells (which are overall hydrophobic, hyphae more than yeast [29]) not due to the chemical interactions, but rather because of their physical properties, such as the topology of the surface. Furthermore, Figure 3g shows the magnified rarely observed *C.a.* yeast cells on the surface of the composite with three percent of UA. These cells were not observed in the hyphal form, and more importantly the visible loss of yeast-cells’ integrity suggests that the mechanism of activity of UA is fungicidal. The deformed morphology of the surface of *C.a.* cells observed by HR SEM has been reported in the presence of only one millimolar UA [21], suggesting that the concentration of UA at the surface of the composite with three percent of UA was minimum one millimolar. This is a reasonable suggestion, because the concentration of three percent UA in PMMA-UA solid was 190 mM, and thus one millimolar UA at its surface would represent a release of only 0.5% UA. Similarly, for some other unsaturated fatty acids (polyunsaturated with C18 to C22 in contrast to UA, which is monounsaturated with C11) has been reported to cause nuclear condensation and fragmentation and thus apoptosis of *C.a.* yeast cells [30].

Moreover, rare *C.a.* cells could be seen on the surface of the composites with 6%, 9% and 12% of UA (Figure 4a–c). This is consistent with the above discussed relatively similar both water contact angles and surface topology. In all, clearly less *C.a.* cells as yeast were observed on the surface of composites with any UA concentration as compared to the native PMMA surface. This observation suggests that the surface of the composites prevent the attachment of *C.a.* cells, i.e., that the PMMA-UA surfaces are *C.a.* repellent. These results showed that our material both: (a) prevent the attachment of *C.a.* cells and (b) have fungicidal effect, because the rare sessile *C.a.* cells were often dead.

**Figure 5:**
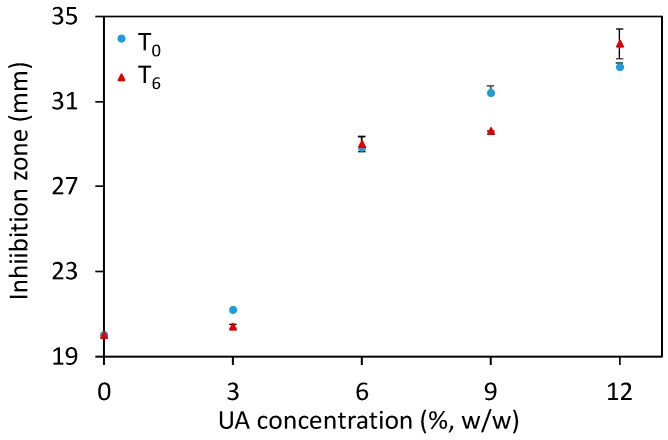
The inhibition zone (measured at two timepoints: after the composites’ preparation, T_0_, and six days after that, T_6_) as a function of the undecylenic acid (UA) concentration in the studied composites (all values are given as mean ± standard deviation).

An additional method to study the antifungal potential of materials is the Kirby–Bauer test (sometimes referred to as a disk diffusion method), where a disk of the studied material is placed on the surface of an agar plate seeded with microorganisms, here *C.a.* cells. After 48 h of incubation at 37 °C, the inhibition zone comprising the disk and the clear region (where *C.a.* growth was inhibited) was measured for the different composites after their preparation (T_0_) and six days after their preparation (T_6_). The results (Figure 5 and Supplementary Figure S1) showed an increase of the inhibition zone along with increasing UA concentration, especially for composites with ≥6% of UA. Moreover, the results were similar in-between the studied time points. These results show that our composites prevent the growth of *C.a.* cells in the vicinity of the composites. Moreover, these results are in agreement with the above given results and confirm the anti-*Candida* activity of the developed composites against the sessile *C.a.* cells. However, the HR SEM study and the Kirby–Bauer test should be complemented by an additional quantitative test.

**Figure 6:**
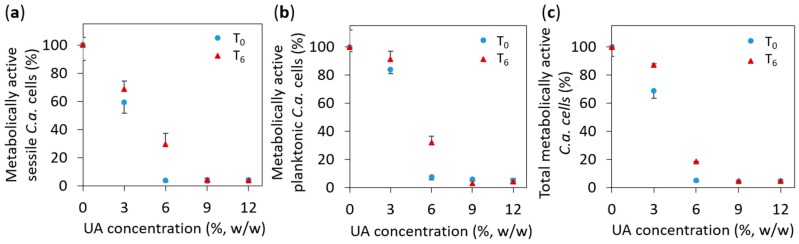
The percentage of metabolically active *Candida albicans* (*C.a.*) cells: (**a**) on the surface of the studied composites (i.e., sessile *C.a.* cells), (**b**) in the supernatant above the composites’ surface (i.e., planktonic *C.a.* cells), and (**c**) in total (that includes both the sessile and the planktonic *C.a.* cells); all values are given as mean ± standard deviation.

Therefore, to quantify the number of *C.a.* cells, which were attached to the surface of the studied materials, we performed an XTT test, which measures the number of metabolically active *C.a.* cells. The XTT test was performed after the composites’ preparation (after 0 days, marked as T_0_) and six days after that (marked as T_6_). The results given in Figure 6a show the relative number of metabolically active *C.a.* cells on the surface of the studied PMMA-UA composites (as compared to the surface of native PMMA). We found that the percentage of metabolically active *C.a.* cells was less than five percent on composites with 9% and 12% of UA as compared to native PMMA for both studied timepoints, i.e., T_0_ and T_6_. In other words, the number of metabolically active *C.a.* cells was reduced by more than 95% in composites with 9% and 12% of UA as compared to native PMMA for both timepoints. On the surface of the composites with six percent of UA, 3.7% and 29.6% of metabolically active *C.a.* cells were measured for T_0_ and T_6_, respectively; while on composites with three percent of UA, the corresponding values were 59.4% and 68.6%, respectively (even these values were significantly different as compared to native PMMA, *p* = 0.001). Overall, the obtained XTT results showed a considerable reduction of the number of metabolically active *C.a.* cells on the surface of PMMA-UA composites in both T_0_ and T_6_. This reduction was more than 95% on the composites: with ≥6% UA in T_0_ and with ≥9% UA in T_6_. Therefore, the developed PMMA-UA composites showed an antifungal surface activity towards *C.a.* sessile cells at an early stage of colonisation (after 24 h), which has not been previously observed in composites of acrylic-denture liners with UA, which was explained by probably low concentrations of the released UA [31]. The low percentages of metabolically active *C.a.* cells measured on the composites’ surfaces are in agreement with the above given HR SEM observation of the PMMA-UA surfaces, where very few *C.a.* cells were observed, often deformed and with loss of their cell integrity (see Figure 3g), i.e., probably metabolically non-active.

Beyond the anti-*Candida* activity of the PMMA-UA surface found against the sessile *C.a.* cells, it was of interest to study if UA molecules from the composites could also affect planktonic *C.a.* cells. This was a challenge, because UA has a limited solubility in water (depending on the temperature and pH, for instance 38.46 mg/L at 20 °C and pH 4.27) and this is reduced in the presence of cell media and *C.a.* cells. In order to study this, we performed an XTT test on the supernatant, which was above the surface of the composites during 24 h incubation with *C.a.* cells. The obtained results (Figure 6b) showed similar values for the planktonic as for the sessile *C.a.* cells. This could be a consequence of the opposing effects of: (a) an expected lower drag-to-cell ratio (drug is here UA) in medium than on the surface of the corresponding composites, and (b) the previously reported lower drag resistance for the planktonic as compared to the sessile *C.a.* cells at the comparable cell concentration [32,33]. Surprisingly, the results given in Figure 6b showed similar results as on the surface of the composites for both studied timepoints. This suggests that our composites affected not only sessile, but also planktonic *C.a.* cells, by reducing the number of metabolically active both sessile and planktonic *C.a.* cells. Therefore, we plotted in Figure 6c the percentages of the total metabolically active *C.a.* cells, which include both the sessile and the planktonic *C.a.* cells, as a function of the UA concentration. We found that the percentage of the total metabolically active *C.a.* cells was less than five percent for composites with 9% and 12% of UA for both timepoints (i.e., percentage of the total metabolically active *C.a.* cells was reduced more than 95%). On the other hand, for composites with 3% and 6% of UA, the percentage of the total metabolically active *C.a.* cells was 68.6% and 5.2%, respectively, after T_0_ (while 87.2% and 18.4%, respectively, after T_6_). This also confirms the above given fungicidal activity of the here developed PMMA-UA composites.

**Figure 7:**
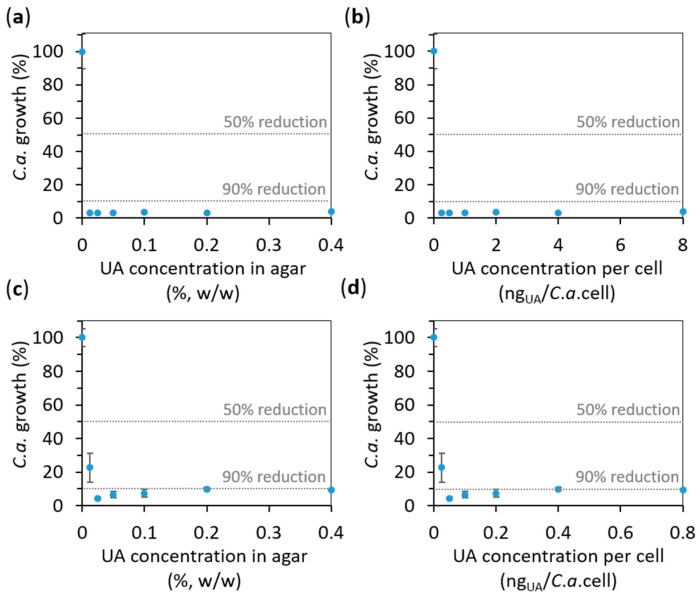
The effect of undecylenic acid (UA) on the *Candida albicans* (*C.a.*) cell growth in the supernatant above the surface of agar containing the given concentrations of UA (evaluated by measuring the absorbance at 620 nm, A_620_). The results obtained for *C.a.* cells concentration of (**a**,**b**) 10^6^ *C.a.* cells/mL and (**c**,**d**) 10^7^ *C.a.* cells/mL are expressed as average percentages of A_620_ readings compared to control (0% UA) versus UA concentration: (**a**,**c**) in agar and (**b**,**d**) per *C.a.* cell calculated for the number of incubated *C.a.* cells (all values are given as mean ± standard deviation). The vertical dashed lines indicate 50% and 90% of the *C.a.* growth reduction.

The effect of UA on the growth of *C.a.* planktonic cells was also studied on agar containing different concentrations of UA (from 0.0125% to 0.4%, see the Materials and Methods for details). Firstly, after the incubation (for 24 h at 37 °C) of *C.a.* cells at a concentration of 10^6^ *C.a.* cells/mL, we measured the absorbance of the supernatant containing the planktonic *C.a.* cells at 620 nm, A_620_. The results expressed as average percentages of A_620_ readings compared to control (0% UA) are given versus UA concentration in agar in Figure 7a. Interestingly, even the lowest UA concentration (0.0125%, *w*/*w*) in agar was sufficient to reduce more than 90% of the *C.a.* planktonic cell growth, i.e., 0.0125% was already the concentration above the one necessary to reduce *C.a.* growth of 90% with respect to the control (called minimal inhibitory concentration (MIC) for 90% growth reduction, i.e., MIC90). Similar results were obtained with the XTT test done on the same suspensions (Supplementary Figure S2). Since we could not measure the dose of UA to which *C.a.* cells are exposed, we calculated the maximal theoretical amount of UA to which a *C.a.* cell could be exposed to in such an experiment (if all UA from the agar would come into the suspension of the incubated *C.a.* cells) by dividing the total mass of UA in the agar substrate by the number of incubated *C.a.* cells. The so-obtained results (Figure 7b) showed that the maximal theoretical concentration could be from 0.25 to 8 ng_UA_/*C.a.* cell for 0.0125% to 0.4% UA in agar, respectively. Since these values seemed to be rather high (the lowest one is above MIC90), we performed the same experiment with ten times higher concentration of *C.a.* cells (10^7^ *C.a.* cells/mL). The results (Figure 7c,d) showed that MIC90 for 10^7^ *C.a.* cells/mL was below the lowest studied concentrations of 0.0125% and 0.025% of UA in agar (corresponding to values between 0.025 and 0.050 ng_UA_/*C.a.* cell). However, even the lowest UA concentration in agar of 0.0125% (i.e., 0.025 ng_UA_/*C.a.* cell) was higher than MIC for 50% growth reduction (MIC50). Thus, only a limit to what MIC90 could be was observed. However, the value for a limit below which is MIC90 found here (0.025%, *w*/*w*, corresponding to 0.025 μg_UA_/mL) is much lower than the 256 μg_UA_/mL, which was previously reported for planktonic *C.a.* cells incubated at 10^7^ *C.a.* cells/mL [31]. We have to note that percentages of the *C.a.* growth in-between samples with the similar drag-to-cell ratios (e.g., 3.1% *C.a.* growth for 0.25 ng_UA_/*C.a.* cell at 10^6^ *C.a.* cells/mL, and 9.9% *C.a.* growth for 0.4 ng_UA_/*C.a.* cell at 10^7^ *C.a.* cells/mL) was typically higher at higher *C.a.* cells’ concentration (10^7^ *C.a.* cells/mL). This is in agreement with the previous reports that the resistance of *C.a.* cells to drags increases with a higher cells’ concentration [32,33]. These results also confirm the fungicidal effect of UA on planktonic *C.a.* cells.

**Figure 8:**
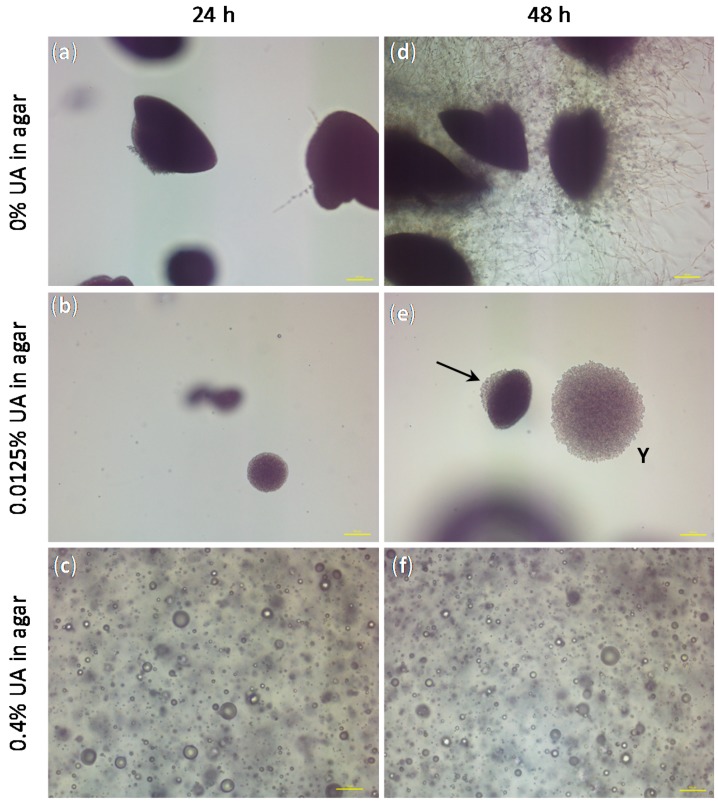
The representative photomicrographs of the *Candida albicans* (*C.a.*) cells imaged after incubation for 24 h (**a**–**c**) and 48 h (**d**–**f**) in the embedded conditions in agar: (**a**,**d**) without undecylenic acid (UA), which served as control, (**b**,**e** spindle-shaped colonies with yeast outgrowth and yeast colonies are indicated by the arrow and Y, respectively) with 0.0125% UA in agar and (**c**,**f**) with 0.4% UA in agar (all scale bars represent 100 μm).

Besides the confirmation of the anti-*Candida* effect of UA on both sessile and planktonic *C.a.* cells, the only study technique that gave us information on the *C.a.* cell type was HR SEM (see Figure 3). Therefore, we performed an embedded filamentation assay for both 24 and 48 h of incubation to have an additional information on the *C.a.* cells development in the presence of UA as compared to the control without UA (representative photomicrographs are given in Figure 8). The concentrations of UA in agar were chosen to be the lowest and the highest values used in the previous test (i.e., 0.0125% and 0.4%). After 24 h of incubation, *C.a.* cells embedded in agar without UA formed spindle-shaped colonies consisting mostly of yeast cells with rare peripheral hyphae and/or pseudohyphae, which typically had lateral yeasts (Figure 8a), as often reported [34,35]. At the same time point, *C.a.* cells embedded in agar with 0.0125% UA formed yeast colonies (smaller than in the control and without hyphae, Figure 8b), while in agar containing 0.4% UA, *C.a.* cells could not be observed, but only spheres probably originating from the leakage of the intracellular material after *C.a.* cells’ death (Figure 8c). After 48 h of incubation, *C.a.* cells embedded in agar without UA formed numerous radially emerging peripheral hyphae, pseudohyphae and lateral yeasts from spindle-shaped yeast colonies (Figure 8a). Moreover, all embedded colonies formed hyphae (since the incubation was at 37 °C) [35]. On the contrary, in agar with 0.0125% of UA, spindle-shaped colonies with yeast outgrowth and yeast colonies (indicated by the arrow and Y in Figure 8e, respectively) could be observed, both without filamentation; while the *C.a.* cells in agar with 0.4% UA observed after 48 h (Figure 8f) looked the same as after 24 h. These results showed fungicidal effect of UA at 0.4% in agar, while a UA concentration of 0.0125% was sufficient to inhibit the yeast-to-hyphae transition and to suppress the *C.a.* growth.

**Figure 9:**
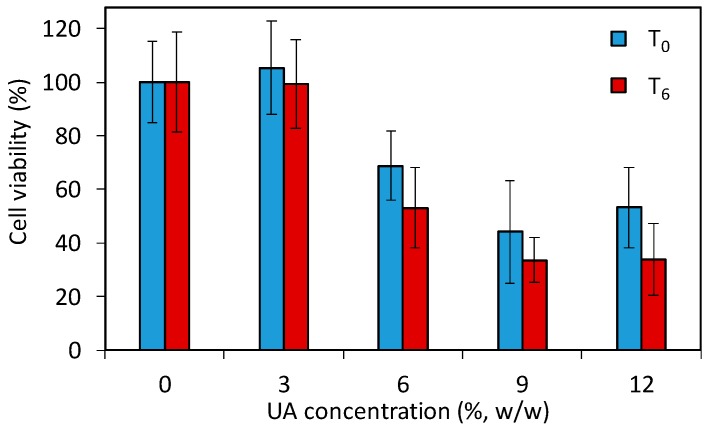
Viability of human A594 cells incubated for 24 h with composite-disc (with different concentrations of undecylenic acid, UA, 3%, 6%, 9% and 12% *w*/*w*) conditioned media measured with the 3-(4,5-dimethylthiazol-2-yl)-5-(3-carboxymethoxypenyl)-2-(4-sulfophenyl)-2*H*-tetrazolium (MTS) test. The cell viabilities are given as percentages of viable cells treated with the composite-disc conditioned media normalized with the number of viable cells treated with control PMMA-disc conditioned media without UA (0% UA). For the two studied time points, T_0_ and T_6_, composites were immersed for 24 h and six days longer, respectively.

The above given study demonstrates that the developed PMMA-UA composites and UA itself are efficient anti-*Candida* agents. However, the use of such composites in patients requires a low toxicity of the antifungal material to human cells. Especially the strong fungicidal effect of UA observed in *C.a.* cells poses the question whether PMMA-UA composites at the here-used UA concentrations (up to 12%) would be toxic to human cells. Therefore, in order to assess the effect of PMMA-UA composites as a final material, we performed a preliminary cytotoxicity study by using the 3-(4,5-dimethylthiazol-2-yl)-5-(3-carboxymethoxypenyl)-2-(4-sulfophenyl)-2*H*-tetrazolium (MTS) test according to the previously reported method [36]. The test (which comprise of 24 h incubation with cells) was performed for two timepoints being six days apart (T_0_ and T_6_) with respect to composites preparation. The results (Figure 9) showed a decrease of the cell viability for composites with six percent UA (68.8% and 53.1% at T_0_ and T_6_, respectively), which was even more pronounced for the composite with nine percent UA (44.2% and 33.6% at T_0_ and T_6_, respectively). Moreover, even at T_0_, meaning zero days after composite preparation and after 24 h immersion of composite-discs in the medium, there was release of UA from composites with greater than six percent, which was sufficient to affect cells. Therefore, composites with 9% and 12% of UA cannot be used as antifungal materials in or on patients due to their toxic effects on human cells (i.e., less than 50% of cells remained viable). However, based on the here-obtained values for cells viability in the preliminary toxicity test, PMMA-UA composites with UA concentrations of up to six percent look still promising for such applications.

So far, there have been reported only a very few studies of one commercial acrylic denture liner (i.e., Coe Soft, GC America, Alsip, IL, USA), which according to the authors contain UA (70 mM UA [29], or one to five percent UA [37], or non-specified UA concentration [31]), but according to the producer this product contains zinc undecylenate [38]. Moreover, it is difficult to compare our results with their results due to the numerous differences in the study conditions (*C.a.* strain, inoculum concentration, temperature and time of incubation, planktonic or sessile cells, etc.). McLain et al. reported a sevenfold reduction of the *C.a.* germ-tube formation without an effect on the growth rate in the presence of 10 μM UA (as compared to control) (*C.a.* strain ATCC 28367 at concentration 10^6^ *C.a.* cells/mL incubated at 39 °C for two hours) [29]. In the same study, the authors reported that on the surface of Coe Soft all adhered *C.a.* cells were yeast form [29]. Although the ability of *C.a.* cells to change their form was suppressed, the adherence of *C.a.* cells and their growth were not inhibited. However, one other study reported the reduction of a *C.a.* biofilm formation, but a higher number of hyphae on Coe Soft as compared to the PMMA surface (*C.a.* strain ATCC 90028 at concentration 10^7^ *C.a.* cells/mL incubated at 37 °C for 24 h) [37]. A more recent study reported that UA released from Coe Soft was appropriate to significantly affect mature *C.a.* biofilms (48 and 72 h) by reducing both the cell counts and their metabolic activity, but not sufficient to influence *C.a.* at an early stage of the colonization, i.e., 24 h (*C.a.* strain ATCC 90028 at concentration 10^7^ *C.a.* cells/mL incubated at 37 °C for 24–72 h) [31]. The same study found a minimal inhibitory concentration required to inhibit *C.a.* planktonic cells growth at concentration of released UA of 256 μg/mL; but at the same concentration of released UA, the fungistatic effect on *C.a.* growth was shown only until eight hours [31]. None of these reports studied the potential toxic effects of UA on human cells. However, beyond this one studied commercial acrylate containing either zinc undecylenate or UA, no new antifungal materials have been developed by incorporation of UA into some commercial acrylate for medical application (especially for dentistry).

Here, we have developed new anti-*Candida* materials by incorporation of UA into commercial PMMA, which allowed UA to be released in a sufficient amount to show simultaneously the inhibition of the adherence at early stage of the colonization (24 h), morphogenesis (yeast-to-hyphae transition) and growth of both sessile and planktonic *C.a.* cells. Therefore, we here present several pieces of evidence that novel PMMA-UA composites have strong fungistatic and fungicidal anti-*Candida* effect. We have also shown that beyond fungicidal effect, UA at larger concentrations (as in composites with ≥9% of UA) can be cytotoxic for human cells, and thus, care has to be taken when UA is used in or on patients.

By taking into consideration global health issues with fungal infections [1,2,3], this study shows potential in combating fungal infections, especially by *C.a.*, thanks to the development of novel fungicidal and/or fungistatic materials in such a way that a sufficient amount of the active antifungal component can reach both sessile and planktonic fungal cells.

## 3. Materials and Methods

### 3.1. Composites Preparation

Commercial Triplex Cold (IvoclarVivadent, Schaan, Liechtenstein) components, solid (polymethyl methacrylate, PMMA) and liquid (mostly methyl methacrylate, MMA) were mixed according to the manufacturer’s instructions (the mixing ratio corresponding to 13 g of solid on 10 mL of the liquid component). Undecylenic acid (UA) (Sigma-Aldrich, St. Louis, Missouri, USA, CAS Number 112-38-9) was physically incorporated into the polymer to obtain 3%, 6%, 9% and 12% (*w*/*w*) of UA in the final composite materials. The so-obtained composites were prepared in the different form according to the tests’ requirements either as free-standing solid disks (Ø 20 mm, prepared in Teflon molds; for contact angle measurements, ATR-FTIR, HR SEM and Kirby–Bauer test) or as solid disks on the bottom of the wells in the 24-well plates (for biofilm formation and XTT assay). In all experiments, the samples with 0% of UA were used as controls. For all experiments, the as-prepared composites were sterilized under the UV-C lamp for 15 minutes prior to any study.

### 3.2. Physico-Chemical Characterization

#### 3.2.1. Fourier Transform Infrared (FTIR) Spectroscopy

FTIR spectra of the studied composites were obtained with the Spectrum One spectrometer (series: 69288, Perkin Elmer, Schwerzenbach, Switzerland). Transmittance from 4600 cm^−1^ to 400 cm^−1^ were given as the average of 64 scans for each curve with a resolution of 4.00 cm^−1^.

#### 3.2.2. Water Contact Angle

The water contact angle on the surface of the composites was measured by the sessile drop method (by EasyDrop Standard, Krüss, Hamburg, Germany, with a monochrome interline CCD camera) using double distilled water (17.6 MΩ; 20 μL; room temperature). All measurements were performed minimum in triplicates. 

### 3.3. Microorganism and Culture Conditions

In this study, we used *C.a.* ATCC90028 strain (Microbiologics, 0264P). *C.a.* stock was kept at −80 °C and after recovery, kept on Sabouraud 4% Glucose Agar (SGA; Sigma-Aldrich 84088) and stored at 4 °C during the experiments. For the Kirby–Bauer test, the determination of the minimum inhibitory concentration and the filamentation assay: the strain was sub-cultured on SGA for 24 h at 37 °C and an inoculum was prepared from freshly grown colonies on SGA at a concentration of 10^6^ *C.a.* cells/mL in 0.9% sterile NaCl (Sodium chloride, 99.5%; Acros, 44730-2500 autoclaved at 121 °C for 20 min). For HR SEM analysis, the determination of the minimum inhibitory concentration and XTT assay: an inoculum was adjusted to a concentration of 10^6^ *C.a.* cells/mL in RPMI 1640 medium (Sigma-Aldrich, R6504-10x1L).

### 3.4. Antifungal Characterization of Composites

#### 3.4.1. High Resolution Scanning Electron Microscope (HR SEM)

Sample preparation: 100 μL of *C.a.* (at concentration 10^6^ *C.a.* cells/mL) placed on the surface of the composite disks were incubated at 37 °C for for 24 h. After the incubation, the *C.a.* suspension was removed, and the composite discs were washed with sterile PBS and dried naturally. After drying, the composite discs were coated with 3 nm thick Iridium coating (Q150t ES from Quorum Technology, Lewes, UK) and as such studied by high resolution Scanning Electron Microscope, HR SEM (high resolution Field Emission-Scanning Electron Microscopes MERLIN from Carl Zeiss Microscopy, Oberkochen, Germany) operating at an acceleration voltage of 3 kV.

#### 3.4.2. Kirby–Bauer Test

*C.a.* cells (at concentration 10^6^ *C.a.* cells/mL) were spread onto Yeast Extract Peptone Dextrose agar (YPD-agar, BD™ 242720) plates (Petri dishes filed in with YPD-agar). The studied PMMA-UA composite disks were placed on the YPD-agar plates seeded by *C.a.* and incubated at 37 °C for 48 h. After incubation, the inhibition zones were obtained by measuring the diameter, which include the disk (dimeter 20 mm) and the clear zone with inhibited *C.a.* growth (minimum of 5 measurements per disk and a minimum of 3 disks per UA concentration were studied). Kirby–Bauer test was performed at two timepoints: after the composites’ preparation (at 0 days, T_0_) and six days after the preparation of the composites (T_6_).

#### 3.4.3. XTT Assay

The preparation of XTT/menadione solution: For the XTT (2,3-bis(2-methoxy-4-nitro-5-sulfo-phenyl)-2*H*-tetrazolium-5-carboxanilide) reduction assay, the XTT (Cayman, CAS 111072-31-2,) saturated solution at 0.5 g/L was prepared in sterile PBS (Dulbecco’s Phosphate Buffered Saline, Zen-Bio, Inc., Research Triangle Park, NC, USA, DPBS-1000), sterilized by filtration using a 0.22 μm pore-size filter, aliquoted into working volumes, and stored at −20 °C when not used. The stock of the XTT solution was thawed before every assay, and the menadione (Cayman, CAY15950-25g) solution previously prepared in the acetone as a 10 mM stock solution was added to the XTT to have a final menadione concentration of 1 μM. The so-obtained solution will be referred to as XTT/menadione.

*C.a*. incubation with composites for XTT assay: 400 μL of a *C.a*. inoculum (at a concentration 10^6^ *C.a.* cells/mL in RPMI 1640 medium) was added to the wells (in 24-well plates), which contained the studied composite disks at the wells’ bottom (disks with 0% of UA were used as controls). The so-prepared well plates were covered with a sterile seal plate film, covered with a lid, sealed with parafilm and incubated for 24 h at 37 °C. After incubation, two different XTT assays were performed:

(a) XTT assay on *C.a.* sessile cells: After 24 h of *C.a.* incubation, the supernatant (liquid above the biofilm) was removed and wells were washed with sterile PBS to remove the remained non-adherent planktonic cells in the wells. After draining these plates in an inverted position, 200 μL the XTT/menadione was added to every well. The plates were covered with a lid, sealed with parafilm, wrapped with aluminum foil and incubated in the dark for 3 h at 37 °C.
(b) XTT assay on *C.a.* planktonic cells: After 24 h of *C.a.* incubation, 200 μL of supernatant (liquid above the sessile cells) was taken from each well and transferred in new 24-well plates. Subsequently, 200 μL the XTT/menadione was added to each of these wells. The plates were covered with a lid, sealed with parafilm, wrapped with aluminum foil and incubated in the dark for 3 h at 37 °C.

XTT measurement: The absorbance of the so-obtained solutions (100 μL placed in the 96-well plate) was measured with a microtiter plate reader (TECAN Infinite M200, Tecan, Männedorf, Switzerland) at 490 nm. As a control, RPMI medium was tested under the same conditions. From the obtained absorbance values, the percentage of metabolically active *C.a.* cells (%) was calculated with the following equation:

percentage of metabolically active *C. a.* cells (%) = [*Abs*(*C.a*.; PMMA-UA) − *Abs*(medium)]/[*Abs*(*C.a*.; PMMA) − *Abs*(medium)]·100% (1)

where *Abs*(*C.a*.; PMMA-UA), *Abs*(*C.a*.; PMMA) and *Abs*(medium) denote the absorbance of liquid containing XTT: with *C.a.* for PMMA-UA composites and PMMA, and without *C.a*. for RPMI medium, respectively.

Both XTT assays (on *C.a.* sessile and *C.a.* planktonic cells) were performed at two timepoints: after the composites’ preparation (at 0 days, T_0_) and six days after the preparation of the composites (T_6_).

### 3.5. Antifungal Characterization of UA

#### 3.5.1. UA Dispersions

UA dispersions in YPD-agar were prepared according to a previously described protocol [39,40] to have six different weight concentrations of UA in dispersions: 0.0125%, 0.025%, 0.05%, 0.1%, 0.2% and 0.4%. After autoclaving and natural cooling down to approximately 40 °C, 40 mL of YPD-agar was added into 50 mL Polypropylene flat falcon tubes (Falcon 62.559.001) containing suitable amount of UA. The so-obtained UA dispersions in YPD-agar were used for the following two tests (given in Section 3.5.2 and Section 3.5.3).

#### 3.5.2. Minimum Inhibitory Concentration (MIC)

One millilitre of dispersion (as described in the previous Section 3.5.1) per well was transfer into a 24-well plate. After solidification of these dispersions inside the wells, 500 μL of *C.a.* suspension (density 10^6^ *C.a.* cells/mL) was added and incubated at 37 °C for 24 h. Upon incubation, MIC was determined by two tests: (a) by measuring the absorbance of 50 μL of the so-obtained *C.a.* suspensions at 620 nm, using a microplate reader (TECAN Infinite M200, Tecan, Männedorf, Switzerland), and (b) by measuring the absorbance at 490 nm (with the same microplate reader) of 50 μL of the so-obtained *C.a.* suspensions where 50 μL of XTT/menadione was added after their incubation in the dark for 3 h at 37 °C.

#### 3.5.3. Embedded Filamentation Assay

In the 5 mL of the two UA dispersions (as described in the previous Section 3.5.1) with UA concentration of 0.0125% and 0.4%, 100 μL of *C.a.* (concentration 10^6^ *C.a.* cells/mL) were added, mixed and poured into sterile Petri dishes (diameter 30 mm). After natural cooling down, Petri dishes were incubated at 37 °C and studied by optical microscope (Nikon Eclipse Ti-E inverted microscope, Nikon Instruments Europe BV, Amsterdam, Netherlands) after 24 h and 48 h. Photomicrographs were taken through the agar matrix.

### 3.6. Cytotoxicity Study of PMMA-UA Composites

#### Toxicity Test

For the 3-(4,5-dimethylthiazol-2-yl)-5-(3-carboxymethoxypenyl)-2-(4-sulfophenyl)-2*H*-tetrazolium (MTS) toxicity test, we used a modified protocol from ref. [36]. In brief, “composite-disc conditioned media” was prepared by immersing a series of PMMA-UA composite discs in PBS (2 discs per composite per 10 mL of PBS) and incubating them for 24 h at 37 °C followed by 10 s of shaking in vortex. For the two studied time points, T_0_ and T_6_, the composites were immersed for 24 h 0 days and 6 days after their preparation, respectively (in other words, composites for T_0_ and T_6_ were immersed in total: 1 day and 7 days, respectively). Human A594 cells were cultured in RPMI-1640 medium (Sigma-Aldrich) supplemented with 10% foetal bovine serum and 2% 5000 U mL^−1^ Penicillin, 5 mg mL^−1^ Streptomycin and 10 mg mL^−1^ Neomycin (Sigma-Aldrich). Four thousand A549 cells per well were cultured in 96-well plates at 37 °C for 24 h, and afterwards exposed to 100 μL media containing 50% fresh growth media and 50% (*v*/*v*) the “disc conditioned media” for an additional 24 h. Cells treated only with medium, with 50% of growth medium and 50% of PBS without disc immersion, as well as with 50% of growth medium and 50% of PBS with PMMA disc immersion served as controls. After 24 h incubation, the supernatant of each well was removed. 100 μL of MTS solution (CellTiter 96^®^ AQueous One Solution Cell Proliferation Assay from Promega, diluted 6 times in medium) was added to the cells. After 2 h incubation in the dark, the absorbance of the formazan product was measured with a microplate reader (Tecan Infinite M200, Tecan, Männedorf, Switzerland) at a wavelength of 490 nm. All experiments were performed in four repetitions. Results are given as means (with standard deviations) of the values obtained in these four repetitions.

## Supplementary Materials

Supplementary materials can be found at www.mdpi.com/1422-0067/19/1/184/s1.
