# Supplementary material for: Fungicidal PMMA-Undecylenic Acid Composites
Source: Int J Mol Sci. 2018 Jan 8;19(1):184. doi: 10.3390/ijms19010184 (PMC5796133; doi:10.3390/ijms19010184)
Supplement: Supplementary file 1 [file ijms-19-00184-s001.pdf]

Supplementary Material

Fungicidal PMMA-undecylenic acid composites

Milica Petrović [1,2], Debora Bonvin [1], Heinrich Hofmann [1] and Marijana Mionić Ebersold [1,*]

[1] Powder Technology Laboratory, Institute of Materials, Ecole Polytechnique Fédérale de Lausanne, Switzerland; debora.bonvin@gmail.com, heinrich.hofmann@epfl.ch, marijanamionic@gmail.com

[2] Faculty of Medicine, University of Nis, Serbia; petrovicmilica21@gmail.com

* Correspondence: marijanamionic@gmail.com; Tel.: +41-76-238-1669

Supplementary Figures

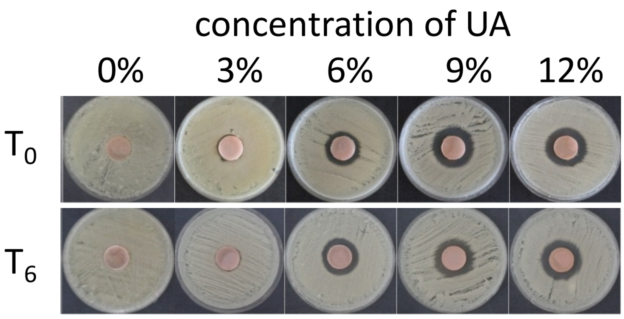

Figure S1. The representative results of Kirby-Bauer tests are given for both studied time points (after composites preparation, T_0, and six days after that, T_6) for the studied composites with the indicated undecylenic acid (UA) concentration.

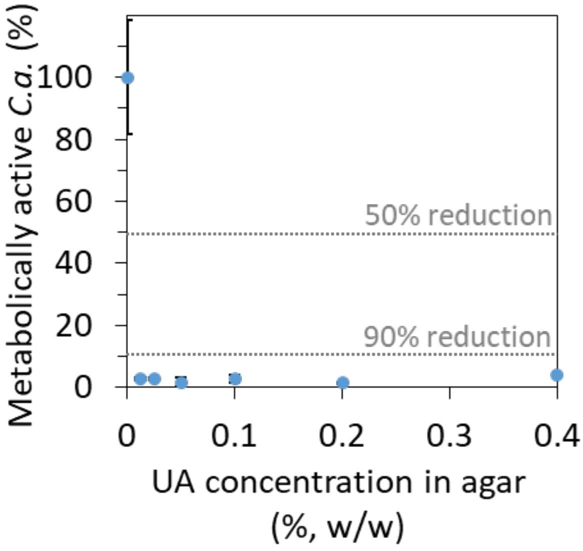

Figure S2. The percentage of metabolically active *Candida albicans* (*C.a.*) cells on the surface of the studied composites (i.e. sessile *C.a.* cells) *versus* undecylenic acid (UA) concentration in agar (all values are given as mean ± standard deviation). The vertical dashed lines indicate 50 and 90% of the *C.a.* growth reduction.
